# Supplementary material for: HIVprotI: an integrated web based platform for prediction and design of HIV proteins inhibitors
Source: J Cheminform. 2018 Mar 9;10:12. doi: 10.1186/s13321-018-0266-y (PMC5845081; doi:10.1186/s13321-018-0266-y)
Supplement: Supplementary file 2 — Additional file 2. Source code of HIVProtI web server. [file 13321_2018_266_MOESM2_ESM.zip › HIVprotI_Source-code/hivprotI/weka-3-6-6/doc/weka/gui/arffviewer/ArffSortedTableModel.html]

ArffSortedTableModel


---


|  |  |  |
| --- | --- | --- |
| **Overview** \| **Package** \| **Class** \| **Tree** \| **Deprecated** \| **Index** \| **Help** \| **Weka's home** | |  |
| **PREV CLASS**   **NEXT CLASS** | **FRAMES**    **NO FRAMES**     **All Classes** |  |
| SUMMARY: NESTED \| FIELD \| CONSTR \| METHOD | DETAIL: FIELD \| CONSTR \| METHOD |  |


---


## weka.gui.arffviewer Class ArffSortedTableModel

```
java.lang.Object
  javax.swing.table.AbstractTableModel
      weka.gui.SortedTableModel
          weka.gui.arffviewer.ArffSortedTableModel
```

**All Implemented Interfaces:**: java.io.Serializable, java.util.EventListener, javax.swing.event.TableModelListener, javax.swing.table.TableModel, Undoable

---

``` public class ArffSortedTableModel extends SortedTableModel implements Undoable ```

A sorter for the ARFF-Viewer - necessary because of the custom CellRenderer.

**Version:**
:   $Revision: 1.4 $

**Author:**
:   FracPete (fracpete at waikato dot ac dot nz)

**See Also:**: Serialized Form

---

**Nested Class Summary**

**Nested classes/interfaces inherited from class weka.gui.SortedTableModel**

`SortedTableModel.SortContainer`


**Constructor Summary**

`ArffSortedTableModel(Instances data)`
  
          initializes the sorter w/o a model, but uses the given data to create
a model from that

`ArffSortedTableModel(java.lang.String filename)`
  
          initializes the sorter w/o a model, but loads the given file and creates
from that a model

`ArffSortedTableModel(javax.swing.table.TableModel model)`
  
          initializes the sorter with the given model


| **Method Summary** | |
| --- | --- |
| `void` | `addTableModelListener(javax.swing.event.TableModelListener l)`             adds a listener to the list that is notified each time a change to data model occurs |
| `void` | `addUndoPoint()`             adds an undo point to the undo history |
| `void` | `attributeAsClassAt(int columnIndex)`             sets the attribute at the given col index as the new class attribute |
| `boolean` | `canUndo()`             returns whether an undo is possible, i.e. |
| `void` | `clearUndo()`             removes the undo history |
| `void` | `deleteAttributeAt(int columnIndex)`             deletes the attribute at the given col index |
| `void` | `deleteAttributes(int[] columnIndices)`             deletes the attributes at the given indices |
| `void` | `deleteInstanceAt(int rowIndex)`             deletes the instance at the given index |
| `void` | `deleteInstances(int[] rowIndices)`             deletes the instances at the given positions |
| `Attribute` | `getAttributeAt(int columnIndex)`             returns the attribute at the given index, can be NULL if not an attribute column |
| `int` | `getAttributeColumn(java.lang.String name)`             returns the column of the given attribute name, -1 if not found |
| `Instances` | `getInstances()`             returns the data |
| `double` | `getInstancesValueAt(int rowIndex, int columnIndex)`             returns the double value of the underlying Instances object at the given position, -1 if out of bounds |
| `java.lang.Object` | `getModelValueAt(int rowIndex, int columnIndex)`             returns the value at the given position |
| `int` | `getType(int columnIndex)`             returns the TYPE of the attribute at the given position |
| `int` | `getType(int rowIndex, int columnIndex)`             returns the TYPE of the attribute at the given position |
| `boolean` | `isMissingAt(int rowIndex, int columnIndex)`             checks whether the value at the given position is missing |
| `boolean` | `isNotificationEnabled()`             returns whether the notification of changes is enabled |
| `boolean` | `isReadOnly()`             returns whether the model is read-only |
| `boolean` | `isUndoEnabled()`             returns whether undo support is enabled |
| `void` | `notifyListener(javax.swing.event.TableModelEvent e)`             notfies all listener of the change of the model |
| `void` | `removeTableModelListener(javax.swing.event.TableModelListener l)`             removes a listener from the list that is notified each time a change to the data model occurs |
| `void` | `renameAttributeAt(int columnIndex, java.lang.String newName)`             renames the attribute at the given col index |
| `void` | `setInstances(Instances data)`             sets the data |
| `void` | `setNotificationEnabled(boolean enabled)`             sets whether the notification of changes is enabled |
| `void` | `setReadOnly(boolean value)`             sets whether the model is read-only |
| `void` | `setUndoEnabled(boolean enabled)`             sets whether undo support is enabled |
| `void` | `sortInstances(int columnIndex)`             sorts the instances via the given attribute |
| `void` | `undo()`             undoes the last action |

**Methods inherited from class weka.gui.SortedTableModel**

`addMouseListenerToHeader, getActualRow, getColumnClass, getColumnCount, getColumnName, getModel, getRowCount, getValueAt, isCellEditable, isSorted, setModel, setValueAt, sort, sort, tableChanged`

**Methods inherited from class javax.swing.table.AbstractTableModel**

`findColumn, fireTableCellUpdated, fireTableChanged, fireTableDataChanged, fireTableRowsDeleted, fireTableRowsInserted, fireTableRowsUpdated, fireTableStructureChanged, getListeners, getTableModelListeners`

**Methods inherited from class java.lang.Object**

`equals, getClass, hashCode, notify, notifyAll, toString, wait, wait, wait`

**Constructor Detail**

### ArffSortedTableModel

```
public ArffSortedTableModel(java.lang.String filename)
```

:   initializes the sorter w/o a model, but loads the given file and creates
    from that a model

    **Parameters:**: `filename` - the file to load

---


### ArffSortedTableModel

```
public ArffSortedTableModel(Instances data)
```

:   initializes the sorter w/o a model, but uses the given data to create
    a model from that

    **Parameters:**: `data` - the data to use

---


### ArffSortedTableModel

```
public ArffSortedTableModel(javax.swing.table.TableModel model)
```

:   initializes the sorter with the given model

    **Parameters:**: `model` - the model to use


**Method Detail**

### isNotificationEnabled

```
public boolean isNotificationEnabled()
```

:   returns whether the notification of changes is enabled

    :   **Returns:**: true if notification of changes is enabled

---


### setNotificationEnabled

```
public void setNotificationEnabled(boolean enabled)
```

:   sets whether the notification of changes is enabled

    :   **Parameters:**: `enabled` - enables/disables the notification

---


### isUndoEnabled

```
public boolean isUndoEnabled()
```

:   returns whether undo support is enabled

    :   **Specified by:**: `isUndoEnabled` in interface `Undoable`
    :   **Returns:**: true if undo support is enabled

---


### setUndoEnabled

```
public void setUndoEnabled(boolean enabled)
```

:   sets whether undo support is enabled

    :   **Specified by:**: `setUndoEnabled` in interface `Undoable`
    :   **Parameters:**: `enabled` - whether to enable/disable undo support

---


### isReadOnly

```
public boolean isReadOnly()
```

:   returns whether the model is read-only

    :   **Returns:**: true if model is read-only

---


### setReadOnly

```
public void setReadOnly(boolean value)
```

:   sets whether the model is read-only

    :   **Parameters:**: `value` - if true the model is set to read-only

---


### getInstancesValueAt

```
public double getInstancesValueAt(int rowIndex,
                                  int columnIndex)
```

:   returns the double value of the underlying Instances object at the
    given position, -1 if out of bounds

    :   **Parameters:**: `rowIndex` - the row index: `columnIndex` - the column index **Returns:**: the underlying value in the Instances object

---


### getModelValueAt

```
public java.lang.Object getModelValueAt(int rowIndex,
                                        int columnIndex)
```

:   returns the value at the given position

    :   **Parameters:**: `rowIndex` - the row index: `columnIndex` - the column index **Returns:**: the value of the model at the given position

---


### getType

```
public int getType(int columnIndex)
```

:   returns the TYPE of the attribute at the given position

    :   **Parameters:**: `columnIndex` - the index of the column **Returns:**: the attribute type

---


### getType

```
public int getType(int rowIndex,
                   int columnIndex)
```

:   returns the TYPE of the attribute at the given position

    :   **Parameters:**: `rowIndex` - the index of the row: `columnIndex` - the index of the column **Returns:**: the attribute type

---


### deleteAttributeAt

```
public void deleteAttributeAt(int columnIndex)
```

:   deletes the attribute at the given col index

    :   **Parameters:**: `columnIndex` - the index of the attribute to delete

---


### deleteAttributes

```
public void deleteAttributes(int[] columnIndices)
```

:   deletes the attributes at the given indices

    :   **Parameters:**: `columnIndices` - the column indices

---


### renameAttributeAt

```
public void renameAttributeAt(int columnIndex,
                              java.lang.String newName)
```

:   renames the attribute at the given col index

    :   **Parameters:**: `columnIndex` - the index of the column: `newName` - the new name of the attribute

---


### attributeAsClassAt

```
public void attributeAsClassAt(int columnIndex)
```

:   sets the attribute at the given col index as the new class attribute

    :   **Parameters:**: `columnIndex` - the index of the column

---


### deleteInstanceAt

```
public void deleteInstanceAt(int rowIndex)
```

:   deletes the instance at the given index

    :   **Parameters:**: `rowIndex` - the index of the row

---


### deleteInstances

```
public void deleteInstances(int[] rowIndices)
```

:   deletes the instances at the given positions

    :   **Parameters:**: `rowIndices` - the indices to delete

---


### sortInstances

```
public void sortInstances(int columnIndex)
```

:   sorts the instances via the given attribute

    :   **Parameters:**: `columnIndex` - the index of the column

---


### getAttributeColumn

```
public int getAttributeColumn(java.lang.String name)
```

:   returns the column of the given attribute name, -1 if not found

    :   **Parameters:**: `name` - the name of the attribute **Returns:**: the column index or -1 if not found

---


### isMissingAt

```
public boolean isMissingAt(int rowIndex,
                           int columnIndex)
```

:   checks whether the value at the given position is missing

    :   **Parameters:**: `rowIndex` - the row index: `columnIndex` - the column index **Returns:**: true if the value at the position is missing

---


### setInstances

```
public void setInstances(Instances data)
```

:   sets the data

    :   **Parameters:**: `data` - the data to use

---


### getInstances

```
public Instances getInstances()
```

:   returns the data

    :   **Returns:**: the current data

---


### getAttributeAt

```
public Attribute getAttributeAt(int columnIndex)
```

:   returns the attribute at the given index, can be NULL if not an attribute
    column

    :   **Parameters:**: `columnIndex` - the index of the column **Returns:**: the attribute at the position

---


### addTableModelListener

```
public void addTableModelListener(javax.swing.event.TableModelListener l)
```

:   adds a listener to the list that is notified each time a change to data
    model occurs

    :   **Specified by:**: `addTableModelListener` in interface `javax.swing.table.TableModel` **Overrides:**: `addTableModelListener` in class `javax.swing.table.AbstractTableModel`
    :   **Parameters:**: `l` - the listener to add

---


### removeTableModelListener

```
public void removeTableModelListener(javax.swing.event.TableModelListener l)
```

:   removes a listener from the list that is notified each time a change to
    the data model occurs

    :   **Specified by:**: `removeTableModelListener` in interface `javax.swing.table.TableModel` **Overrides:**: `removeTableModelListener` in class `javax.swing.table.AbstractTableModel`
    :   **Parameters:**: `l` - the listener to remove

---


### notifyListener

```
public void notifyListener(javax.swing.event.TableModelEvent e)
```

:   notfies all listener of the change of the model

    :   **Parameters:**: `e` - the event to send to the listeners

---


### clearUndo

```
public void clearUndo()
```

:   removes the undo history

    :   **Specified by:**: `clearUndo` in interface `Undoable`

---


### canUndo

```
public boolean canUndo()
```

:   returns whether an undo is possible, i.e. whether there are any undo points
    saved so far

    :   **Specified by:**: `canUndo` in interface `Undoable`
    :   **Returns:**: returns TRUE if there is an undo possible

---


### undo

```
public void undo()
```

:   undoes the last action

    :   **Specified by:**: `undo` in interface `Undoable`

---


### addUndoPoint

```
public void addUndoPoint()
```

:   adds an undo point to the undo history

    :   **Specified by:**: `addUndoPoint` in interface `Undoable`


---


|  |  |  |
| --- | --- | --- |
| **Overview** \| **Package** \| **Class** \| **Tree** \| **Deprecated** \| **Index** \| **Help** \| **Weka's home** | |  |
| **PREV CLASS**   **NEXT CLASS** | **FRAMES**    **NO FRAMES**     **All Classes** |  |
| SUMMARY: NESTED \| FIELD \| CONSTR \| METHOD | DETAIL: FIELD \| CONSTR \| METHOD |  |


---
